# Catchment characteristics and seasonality control the composition of microbial assemblages exported from three outlet glaciers of the Greenland Ice Sheet

**DOI:** 10.3389/fmicb.2022.1035197

**Published:** 2022-11-29

**Authors:** Kristýna Vrbická, Tyler J. Kohler, Lukáš Falteisek, Jon R. Hawkings, Petra Vinšová, Marie Bulínová, Guillaume Lamarche-Gagnon, Stefan Hofer, Anne M. Kellerman, Amy D. Holt, Karen A. Cameron, Martina Schön, Jemma L. Wadham, Marek Stibal

**Affiliations:** ^1^Department of Ecology, Faculty of Science, Charles University, Prague, Czechia; ^2^River Ecosystems Laboratory, Alpine and Polar Environmental Research Center, Ecole Polytechnique Fédérale de Lausanne (EPFL), Lausanne, Switzerland; ^3^Department of Earth and Environment, University of Pennsylvania, Philadelphia, PA, United States; ^4^Department of Geosciences, UiT, The Arctic University of Norway, Tromsø, Norway; ^5^Bristol Glaciology Centre, School of Geographical Sciences, University of Bristol, Bristol, United Kingdom; ^6^Department of Geosciences, UiO University of Oslo, Oslo, Norway; ^7^Department of Earth, Ocean and Atmospheric Sciences, Florida State University, Tallahassee, FL, United States; ^8^School of Geographical & Earth Sciences, University of Glasgow, Glasgow, United Kingdom

**Keywords:** ecological indicators, subglacial drainage system, glacial hydrology, proglacial stream, 16S rRNA gene

## Abstract

Glacial meltwater drains into proglacial rivers where it interacts with the surrounding landscape, collecting microbial cells as it travels downstream. Characterizing the composition of the resulting microbial assemblages in transport can inform us about intra-annual changes in meltwater flowpaths beneath the glacier as well as hydrological connectivity with proglacial areas. Here, we investigated how the structure of suspended microbial assemblages evolves over the course of a melt season for three proglacial catchments of the Greenland Ice Sheet (GrIS), reasoning that differences in glacier size and the proportion of glacierized versus non-glacierized catchment areas will influence both the identity and relative abundance of microbial taxa in transport. Streamwater samples were taken at the same time each day over a period of 3 weeks (summer 2018) to identify temporal patterns in microbial assemblages for three outlet glaciers of the GrIS, which differed in glacier size (smallest to largest; Russell, Leverett, and Isunnguata Sermia [IS]) and their glacierized: proglacial catchment area ratio (Leverett, 76; Isunnguata Sermia, 25; Russell, 2). DNA was extracted from samples, and 16S rRNA gene amplicons sequenced to characterize the structure of assemblages. We found that microbial diversity was significantly greater in Isunnguata Sermia and Russell Glacier rivers compared to Leverett Glacier, the latter of which having the smallest relative proglacial catchment area. Furthermore, the microbial diversity of the former two catchments continued to increase over monitored period, presumably due to increasing hydrologic connectivity with proglacial habitats. Meanwhile, diversity decreased over the monitored period in Leverett, which may have resulted from the evolution of an efficient subglacial drainage system. Linear discriminant analysis further revealed that bacteria characteristic to soils were disproportionately represented in the Isunnguata Sermia river, while putative methylotrophs were disproportionately abundant in Russell Glacier. Meanwhile, taxa typical for glacierized habitats (i.e., *Rhodoferax* and *Polaromonas*) dominated in the Leverett Glacier river. Our findings suggest that the proportion of deglaciated catchment area is more influential to suspended microbial assemblage structure than absolute glacier size, and improve our understanding of hydrological flowpaths, particulate entrainment, and transport.

## Introduction

Glacial meltwater is highly influential to downstream ecosystems by creating a unique habitat template (Uehlinger et al., 2010), exporting nutrients and organic matter (e.g., Hood et al., 2009; Singer et al., 2012; Hawkings et al., 2015; Kohler et al., 2017; Irvine-Fynn et al., 2021), and dispersing microbial life (Wilhelm et al., 2013; Cameron et al., 2017a; Hotaling et al., 2019; Kohler et al., 2020b; Ezzat et al., 2022). At the same time, glaciers are retreating around the world at unprecedented rates (IPCC, 2019), altering their relative importance as a water source in catchments as the balance of water coming from non-glacial sources, including groundwater and precipitation, changes (Milner et al., 2017; Holt et al., 2021). As a result, sources and relative quantities of solutes, particulates, and microbial cells from within catchments are likely to shift with glacier shrinkage, potentially impacting downstream ecosystems.

The Greenland Ice Sheet (GrIS) represents one of the largest bodies of ice on Earth, and its meltwater bears considerable influence on downstream freshwater and marine ecosystems (Hawkings et al., 2014, 2017). Outlet glaciers of the GrIS are subject to a seasonally dynamic hydrological regime (Chandler et al., 2013; Hawkings et al., 2021), with the subglacial drainage system evolving from an inefficient distributed system with the onset of melt to one that is more efficient and channelized over the summer (Chu, 2014). The hydrologically active catchment area also expands during this time, creating new possibilities for accessing and draining additional supra- and subglacial areas (Kohler et al., 2017). The subsequent mobilization and export of solutes and particulates from the glacial environment represents an important source of nutrients, organic carbon, and microbial cells to downstream ecosystems (Kohler et al., 2017; Cameron et al., 2017a). Yet, the timing and extent of these flowpaths are expected to change with a warming climate, potentially altering the types and relative proportions of materials transported by proglacial streams.

Within the glacial environment, microbial communities differ between distinct habitat types (Hotaling et al., 2017). For example, the supraglacial habitat (at the glacier surface) receives solar radiation, promoting the growth of photoautotrophic organisms such as glacier algae and cyanobacteria (Hodson et al., 2008; Stibal et al., 2012a; Williamson et al., 2020) which in turn sustains heterotrophic organisms including bacteria and fungi (Kaczmarek et al., 2016). In contrast, the subglacial environment (at the glacier bed) is characterized by the complete absence of light and restricted gas availability, which can lead to the development of anoxia (Wadham et al., 2004; Lamarche-Gagnon et al., 2019). Here, active prokaryotic life is represented by facultatively anaerobic heterotrophs utilizing legacy organic carbon substrates (Vinšová et al., 2022) as well as chemolithoautotrophs (Boyd et al., 2011, 2014) and methanogens (Boyd et al., 2010; Stibal et al., 2012b; Dieser et al., 2014). As glaciers seasonally melt, these different habitat types become hydrologically connected (Cameron et al., 2020), and entrained microbial cells are exported from the glacial habitat and into proglacial streams.

While glaciers are a significant source of suspended cells in the water column of proglacial streams, other sources within the hydrological catchment may gain influence downstream. For example, eroding stream banks, inlets of tributary streams, and thawing permafrost may also contribute to the transported “seed bank” (Battin et al., 2016; Hauptmann et al., 2016; Brandani et al., 2022). Over the course of the melt season, the relative importance of these sources may increase as hydrological connectivity intensifies within the catchment (Crump et al., 2003; Hauptmann et al., 2016; Cavaco et al., 2019). Understanding changes in the suspended assemblage structure may help us to understand the extent of this hydrological connectivity within glacier stream catchments, but also to interpret downstream biological changes, especially if transported cells represent potential colonists for benthic biofilms (Cameron et al., 2017b; Ezzat et al., 2022).

Here, we investigated how meltwater microbial assemblages from three rivers draining glaciers of different sizes, and with different glacierized: proglacial catchment ratios, differ between each other and over the hydrological rising limb of a summer melt season. To do this, we collected daily water samples simultaneously from three proglacial streams draining outlet glaciers of the GrIS for approximately 3 weeks over the 2018 summer. We hypothesized that assemblage structure and diversity should differ between the three catchments due to their catchment characteristics, as well as with the expansion of the glacierized and proglacial hydrological catchment areas. Understanding how the composition of microbial assemblages differs between catchments and over the course of the melt season helps us to understand both the temporal shifts in catchment connectivity, as well as downstream ecosystem changes to anticipate in the future.

## Materials and methods

### Study sites

**Figure 1:**
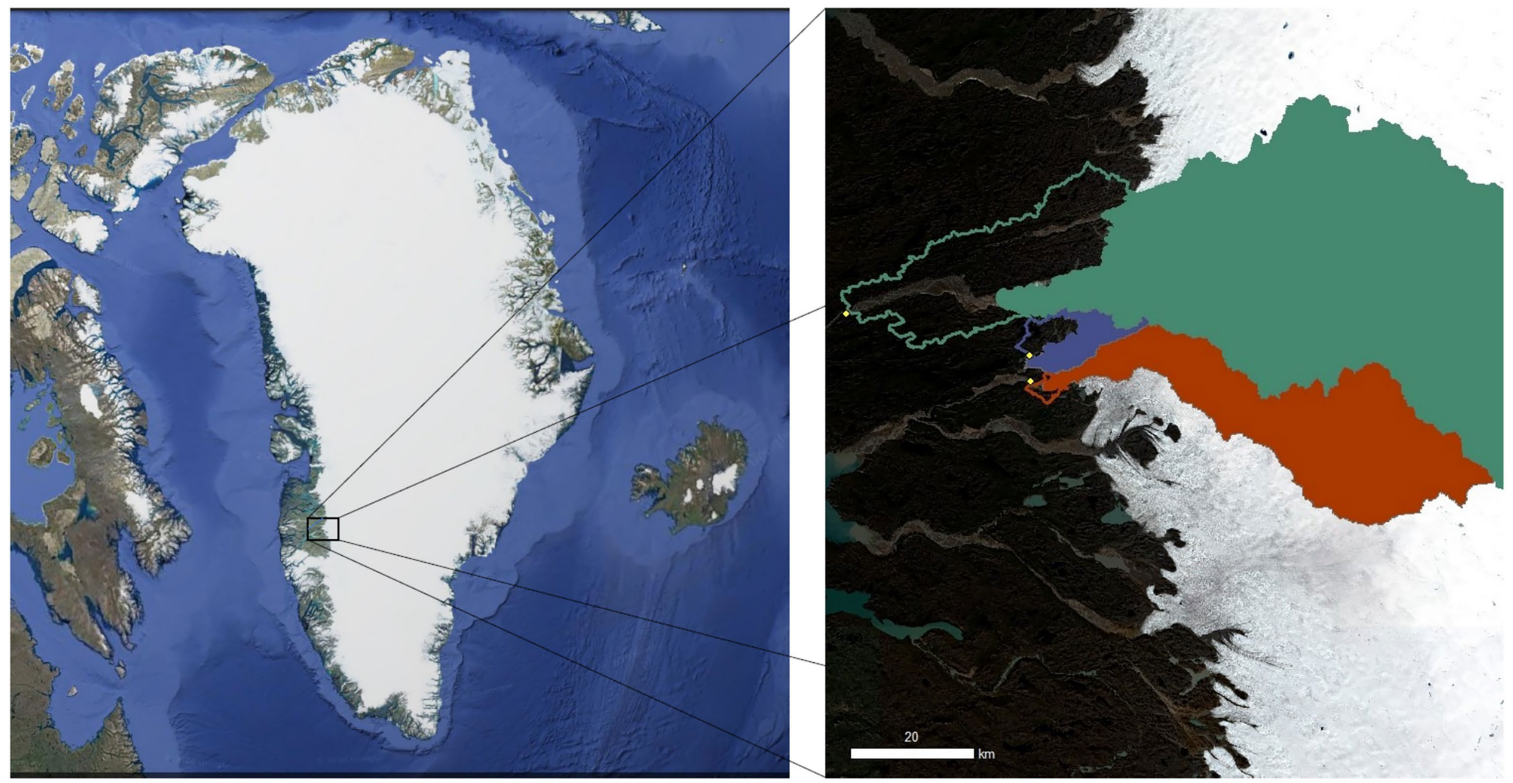
Map of the three Greenland Ice Sheet (GrIS) catchments evaluated in the study. The northernmost green catchment is Isunnguata Sermia (IS), the middle purple catchment is Russell (RG), and the southernmost orange catchment is Leverett (LG). Solid colors indicate the glacierized catchment, while outlines indicate the proglacial catchment area. The left panel is a composite image of Greenland from Landsat (US Geological Survey, via Google), and the right panel was created with Sentinel 2 imagery (Level 2a, 16 August 2020, downloaded from https://earthengine.google.com/) with overlying catchment data from Hawkings et al. (2021) and Mankoff et al. (2020).

Fieldwork was conducted on three proglacial streams draining outlet glaciers of the GrIS, including Isunnguata Sermia (IS), Leverett Glacier (LG), and Russell Glacier (RG), over the 2018 summer (June–July). We report glacierized and proglacial catchment areas as calculated in arcGIS using the methodology outlined in Kohler et al. (2020a) and rounded to two significant digits (Table 1; Figure 1). Briefly, Isunnguata Sermia (IS) represented the largest hydrological catchment and was also the northernmost outlet glacier, reaching up to 120 km into GrIS interior (Lindbäck et al., 2015; Hawkings et al., 2021), and is estimated to be ~14,000 km^2^. For IS, samples were collected from the Isortoq river ~25 km from the glacier terminus (67.166° N, 50.889° W), which was the nearest logistically suitable location, and the farthest from the glacial terminus of the three. As such, IS also had the largest unglaciated catchment area, which we estimated to be ~560 km^2^. Upstream of the sampling site, the river is broad and braided, and has several lateral inputs of tributary streams, with the biggest one being the stream fed by Akuliarusiarssuk Glacier, which flows into the Isortoq River ~8 km from the glacier snout (Figure 1). In this study, we consider the glacial/proglacial catchment area of IS together with the Akuliarusiarssuk Glacier, since both likely serve as important inputs of cells and nutrients, and we collectively refer to them as “IS.”

**Table 1:** Ratios of proglacial catchment sizes (“Proglacial”) and estimated glacier catchment sizes (“Glacier”). In the case of LG’s glacier catchment size, a mean value (840 km^2^) was used.

Site	Proglacial catchment	Glacierized catchment	Proglacial: Glacial	Glacial: Proglacial	Latitude	Longitude
IS	560	14,000	0.04	25	67.166° N	50.889° W
LG	11	840	0.01	76.36	67.066° N	50.215° W
RG	40	81	0.49	2.03	67.104° N	50.217° W

The intermediate-sized glacierized catchment drains LG and is the southernmost of the three sites. The LG glacierized catchment extends ~80 km into the GrIS interior (Cowton et al., 2012; Hawkings et al., 2021), and its area was estimated to be ~840 km^2^. The river exits the glacier through a well-defined portal area and then flows over bedrock with only small inlets. We sampled the LG river close to the glacial terminus (within ~2 km; 67.066° N, 50.215° W). Given the relatively short distance between the sampling site and the glacier terminus, the non-glaciated catchment area was the smallest (estimated to be 11 km^2^) of the studied watersheds, and may therefore bear much less influence in comparison to IS and RG. The chemical and hydrological characteristics of LG have been well-documented in previous work (e.g., Kohler et al., 2017; Lamarche-Gagnon et al., 2019; Hawkings et al., 2021).

The third proglacial stream drains RG and was sampled ~10 km from the glacier terminus (67.104° N, 50.217° W). This glacier is located between IS and LG, and extends ~10–15 km inland (Lindbäck et al., 2015; Hawkings et al., 2021) with an estimated catchment area of 81 km^2^ (Hawkings et al., 2021). The RG proglacial area is comprised of several sub-catchments which are estimated to be ~40 km^2^ in size, and the stream flows alongside the glacier margin for several kilometers. The sampling site was preceded by two small lakes surrounded by developed tundra through which the river flows, as well as a few non-glacial tributaries. Rivers flowing from LG and RG join together downstream to form the Akuliarusiarsuup Kuua (Figure 1) – a major tributary of the Watson River. Similar to LG, RG has also been the subject of numerous studies over the last few decades, and its chemistry, hydrology, and biology are relatively well-characterized (e.g., Dieser et al., 2014; Andrews et al., 2018; Kohler et al., 2020b; Hawkings et al., 2021; Pain et al., 2021).

### Physical and chemical characteristics of streamwater

Physical and chemical analyses of streamwater were conducted as described in Hawkings et al. (2021). Briefly, sensors were installed on stable bedrock sections of each stream to measure stage, turbidity, electrical conductivity (EC), and pH. Discharge and suspended sediment concentrations (SSCs) were calculated based on stage and turbidity, respectively, with standard curves created based on manual measurements as described in Hawkings et al. (2021). Specifically, rhodamine dye injections were used to calibrate the relationship between stage and discharge, and a known volume of raw streamwater was filtered onto pre-weighed nitrocellulose filters on a Nalgene filter tower, dried, and re-weighed to calibrate continuous SSC from turbidity measurements. A subset of these manual measurements of SSC (*n* = 14 for IS and LG, and *n* = 13 for RG) were furthermore used to measure percent total carbon using an elemental analyzer (Elementar vario PYRO cube R^©^, Langenselbold, Hesse, D). The detection limit for carbon was around 0.001% with a coefficient of variation of 2.13% according to 8 replicates of a reference soil standard (Soil Standard 33,840,025, cert. 133,317, C = 2.29%; Elemental Microanalysis Ltd., United Kingdom).

### Microbiological sampling

Water samples were collected for approximately 3 weeks over the 2018 summer coinciding with the hydrological rising limb of the melt season (i.e., preceding peak melt), with one sample collected each day to measure temporal changes in the exported microbial assemblages (Supplementary Table S1).

The sampling was performed between 22 June and 14 July for IS, between 21 June and 13 July at LG, and between 21 June and 15 July at RG. At each stream, one sampling site was chosen to monitor and used every day to collect water samples at the same time. This was 11:00 for RG and LG, and at 15:00 for IS. The delayed timing for IS was due to the sampling site being located further downstream than in the other two, and in order to sample a comparable parcel of water on the stream’s daily hydrograph. Three 24-h sampling campaigns were also conducted, with one sample taken every 2 h over a full diel cycle to explore diurnal patterns in assemblage structure. The 24-h sampling campaigns took place on 24–25 June, 4–5 July, and 10–11 July 2018.

Water for microbiological characterization was filtered directly from the stream using a sterile syringe and was passed through a Sterivex filter (0.22 μm, Millipore, Billerica, MA, United States) until it was clogged (200–600 ml, with most <500 ml). This way, a similar amount of suspended sediment, the primary source of microbial cells, was collected on each filter. Filters were flushed of water, preserved with LifeGuard^®^ preservation solution (MO BIO, Carlsbad, CA, United States), and frozen as soon as possible (within 2 h of collection at −20°C).

### Nucleic acids extraction, sequencing, and bioinformatics analyses

DNA isolation was performed directly on thawed Sterivex filters using the PowerWater® Sterivex™ DNA Isolation Kit (MO BIO) following the manufacturer’s instructions. DNA concentration was measured by using Invitrogen™ Qubit™ 4 Fluorometer with Qubit™ 1X dsDNA HS Assay Kit (Invitrogen, Carlsbad, CA, United States). The V4 region of 16s rRNA gene was PCR amplified using primers 515F (GTGYCAGCMGCCGCGGTAA) and 806R (GGACTACNVGGGTWTCTAAT) and sequenced at SEQme s.r.o. (Dobříš, Czechia) on the Illumina MiSeq platform using 2× 250 bp arrangement.

Sequence data were analyzed through the pipeline SEED v2.1.2 (Větrovský et al., 2018). Paired ends were joined by fastq-join (Aronesty, 2011), and primers cut from the dataset manually. The OTUs were created with 97% similarity, and chimeras were removed using Usearch v.8.1.1861 (Edgar, 2013). The consensus from each OTU was constructed from a MAFFT alignment (Katoh and Standley, 2013). The OTUs were tentatively identified against SILVA nr. 132 database (Quast et al., 2013) in Mothur 1.42.0 (Schloss et al., 2009), while taxonomy was corrected using the NCBI database. Singletons were removed along with chloroplasts, mitochondria, eukaryotic organisms, and OTUs identified as common laboratory contaminants (such as *Escherichia*, *Ralstonia* or *Brevundimonas*). The contaminant taxa were identified by comparing with published lists of known contaminants and our internal database (Salter et al., 2014; Glassing et al., 2016). Overrepresentation of the putative contaminant sequences in GenBank, as well as their origin from varied or irrelevant environments, was used as a supporting criterion in contaminant identification.

Approximately one third of samples had a number of reads << 7,853, and were predominantly from IS and LG during the early part of the melt season, which had high suspended sediment loads which may have interfered with extraction. It was therefore not possible to use all samples due to the low numbers of reads from this subset. The total number of samples used in this study was 112 (IS: *n* = 29; LG: *n* = 29; RG: *n* = 54), and the number of reads per sample ranged from 7,853 to 29,900. The final dataset was accordingly rarefied to 7,853 reads and subsequently transformed to relative abundances. Representative sequences of selected highly abundant OTUs were deposited in GenBank under accession numbers OP279921-OP279953. All sequences have been deposited in the MG-RAST database (Meyer et al., 2008) under accession number mgp104407.

### Statistical analysis

For statistical analysis, all samples available for each catchment (including the 24 h samples) were used together unless stated otherwise. All statistical analyses were performed using R (R Core Team, 2017, version 1.4.1717) and all plots were made with the *ggplot2* R package (Wickham, 2016).

Alpha diversity metrics (number of OTUs, Shannon Diversity) were calculated in the *phyloseq* package (McMurdie and Holmes, 2013). Analysis of Variance (ANOVA) was used to statistically test for differences between glacier stream catchments, followed by pairwise comparisons using Tukey’s Honest Significant Differences (TukeyHSD).

To visualize differences in assemblage structure among the three catchments, principal coordinates analysis (PCoA) on Bray-Curtis distances were performed in the *microeco* package (Liu et al., 2021), which was followed by a heatmap of the 40 most abundant taxa. To test if assemblage structure significantly differed between catchments, PERMANOVA (Anderson, 2001) was performed on Bray-Curtis distances calculated for the full dataset by using *adonis2* in the *vegan* package (Oksanen et al., 2018). To investigate which microbial groups are specifically driving differences between catchments, we further performed linear discriminant analysis effect size (LEfSe; Segata et al., 2011) implemented in the *microeco* package.

To investigate relationships between discharge, EC, pH, TSS, and day of the year (plotted as decimal days, hereafter “DOY”) with individual OTUs, we identified differentially abundant taxa through a random forest analysis using the *randomForest* package (Breiman, 2001) and implemented in R (Liaw and Wiener, 2002). Pearson correlations were calculated and corrected using the false discovery rate correction method. Resulting Pearson correlations were visualized in a heatmap, which was also generated within the *microeco* package. Finally, the OTUs chosen by the random forest analysis were then characterized based on their taxonomy and comparison with habitat preferences of the closest BLAST hits to determine their putative metabolisms and ecology.

Seasonal changes in our most abundant metabolism types as defined by the selected OTUs were also investigated. For this analysis, we used only samples collected each day at the same time (i.e., 24 h samples were omitted). However, if a sample from a particular timepoint was missing, we used a 24 h sample collected within 2 h from the regular sampling time if one was available. Specifically, this concerned two samples of IS from DOY 176 and 186. For the following analysis, we chose all OTUs which were more abundant than 0.99% in at least one sample, which altogether was 33 OTUs. To determine their potential metabolisms, these OTUs were thoroughly compared with the NCBI nt/nr database, while only BLAST hits consistent with the previous identification of the OTUs using the Silva database and, concurrently, belonging to well-characterized isolates, were considered. Most of the abundant OTUs were closely related to isolates characterized by cultivation or genome sequencing. Thus, the basic physiological traits were derived from these microorganisms. We assume that these traits are conserved sufficiently to be adopted for genotypes sharing ca 98–100% homology of their 16S rRNA genes (Langille et al., 2013). In a few cases, the OTUs were placed within groups, where some of the characteristics are significantly varied (e.g., *Comamonadaceae* gen. sp.). These unreliable traits were not used in the analyses. We then chose six metabolism types: methylotrophs, iron oxidizers, heterotrophs and facultative autotrophs, autotrophs, aerobes and facultative anaerobes, and anaerobes. The aerobic lifestyle of *Comamonadaceae* (OTU 5) members was left unresolved due to high SSU rRNA similarity and the co-occurrence of aerobic/anaerobic lifestyles within this group. When analyzing the temporal dynamics, we transformed these data to relative abundances calculated from the sum of the selected OTUs sequences (rather than the full dataset) to reduce the effect of rare taxa which appear later during the season. The significance of seasonal changes in metabolisms was then tested by regression analysis.

## Results and discussion

In this study, we investigated the differences in suspended microbial assemblages between three proglacial streams during a three-week period of the 2018 melt season. These streams differ in the size of their glacierized catchment, but also in the proportion of their glacierized to proglacial catchment areas. Here, we hypothesize that the proportion of catchment type (glacial vs. non-glacial), as well as the stage of the melt season, are likely the major controls explaining differences in the structure of transported assemblages. This work is an important first step in understanding the temporally- and spatially-dynamic hydrological connectivity of proglacial streams to their catchments from glacier to estuary.

### Physical and chemical characteristics of catchments

**Figure 2:**
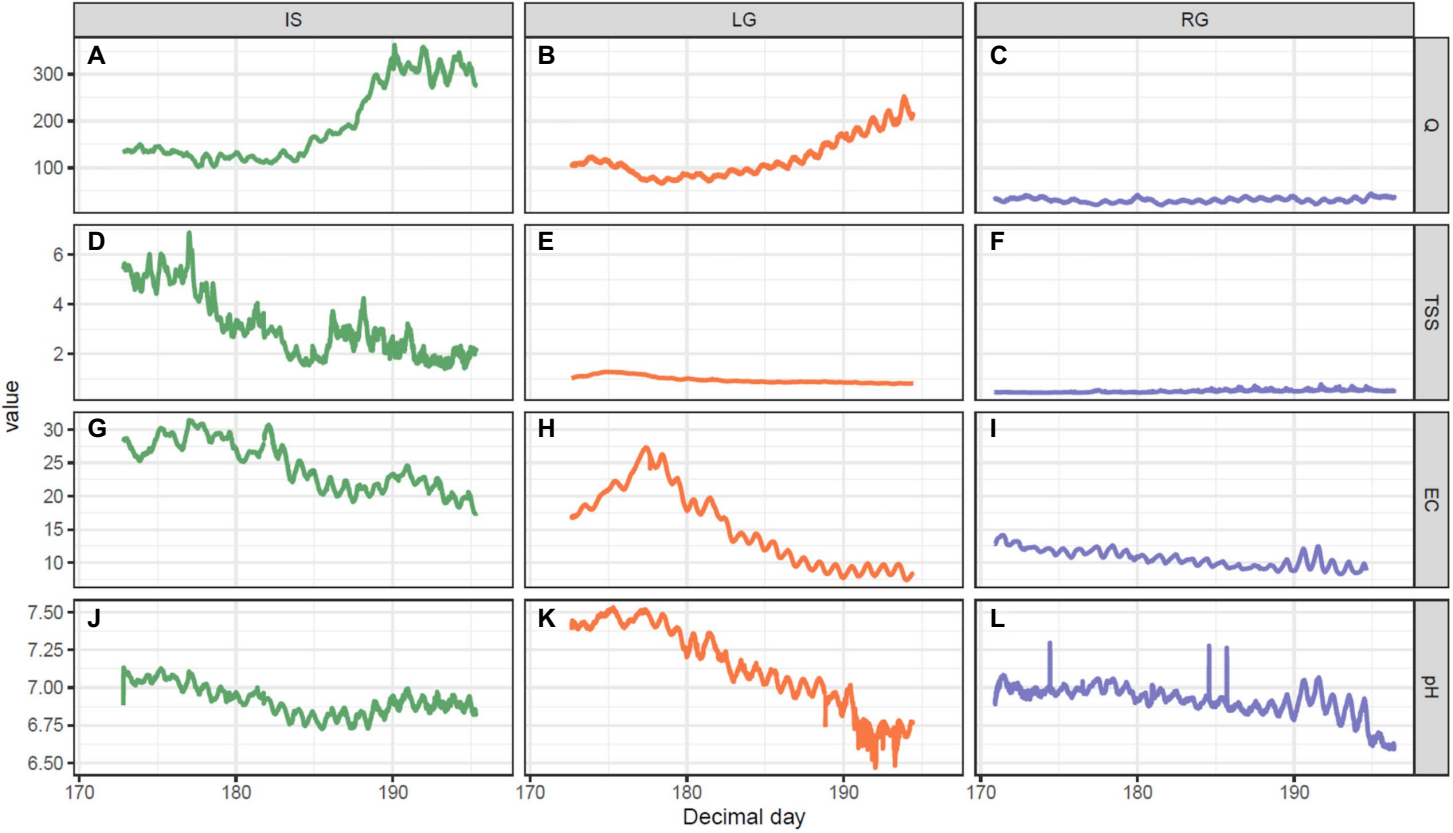
Hydrochemical measurements, including: **(A–C)** Discharge (Q, m^3^ s^−1^), **(D–F)** suspended sediments (TSS, g L^−1^), **(G–I)** electrical conductivity (EC, μS cm^−1^), and **(J–L)** pH over the course of the melt season for the three GrIS outlet glacier catchments over the 2018 summer. The left column is Isunnguata Sermia (IS), middle column Leverett (LG), and right column Russell (RG).

The three proglacial streams differed in terms of their monitored physical and chemical characteristics (Figure 2). For example, discharge at IS (mean = 192 m^3^ s^−1^, range 101–362 m^3^ s^−1^, Figure 2A) was greater than LG (mean = 120 m^3^ s^−1^, range 66–253 m^3^ s^−1^, Figure 2B) and RG (mean = 31 m^3^ s^−1^, range 20–44 m^3^ s^−1^, Figure 2C). In the middle of the sampling period (DOY 185), discharge started to increase at both IS and LG, reaching the highest values at the end of the sampling period. TSS did not show any substantial changes during the season in LG (mean = 0.96 g L^−1^, range 0.79–1.28 g L^−1^, Figure 2E) and RG (mean = 0.5 g L^−1^, range 0.44–0.78 g L^−1^, Figure 2F), compared to IS (mean = 3.13 g L^−1^, range 1.4–6.88 g L^−1^, Figure 2D), which had the highest values and showed a declining trend over the study period. EC decreased at RG (mean = 10.7 μS cm^−1^, range 8.3–14.2 μS cm^−1^, Figure 2I) and IS (mean = 24.6 μS cm^−1^, range 17.4–31.5 μS cm^−1^, Figure 2G) but rose in the first third of the sampling period and then started to decrease at LG (Figure 2H). Lastly, pH was similar at all sites (Figures 2J–L) and showed slight decrease over time, which was most pronounced at LG (mean = 7.1, range 6.1–7.5, Figure 2K).

The size of the glaciated catchment had an influence on the hydrology of these three streams, with the greatest glacierized catchment sizes having the greatest discharge (i.e., IS > LG > RG), and the decrease in TSS and EC over the monitored period indicating solute dilution with increasing discharge as the melt season progressed. The monitored period did not comprise a period of pronounced “outburst” events described in other studies from other seasons investigating these sites (in particular LG; e.g., Chandler et al., 2013, Hawkings et al., 2014, Kohler et al., 2017). “Outburst” events are triggered by the sudden drainage of supraglacial lakes, and are characterized by concomitant peaks in discharge and TSS. The lack of this phenomenon (as demonstrated through sensor data) is likely due to some relatively cool weather during the sampling period, which slowed the progression of melt compared to past seasons that would typically experience peak melt. As a result, our dataset is likely more characteristic of “early season” conditions in this portion of the GrIS that precede the “outburst period”; or, of a hiatus of “outburst periods,” than more characteristic of “late season.” Serendipitously, this lack of outburst events within our field study substantially simplifies interpretation, and allows for more straightforward comparisons of stream assemblages in the context of their catchments.

### Assemblage diversity and structure between catchments

**Figure 3:**
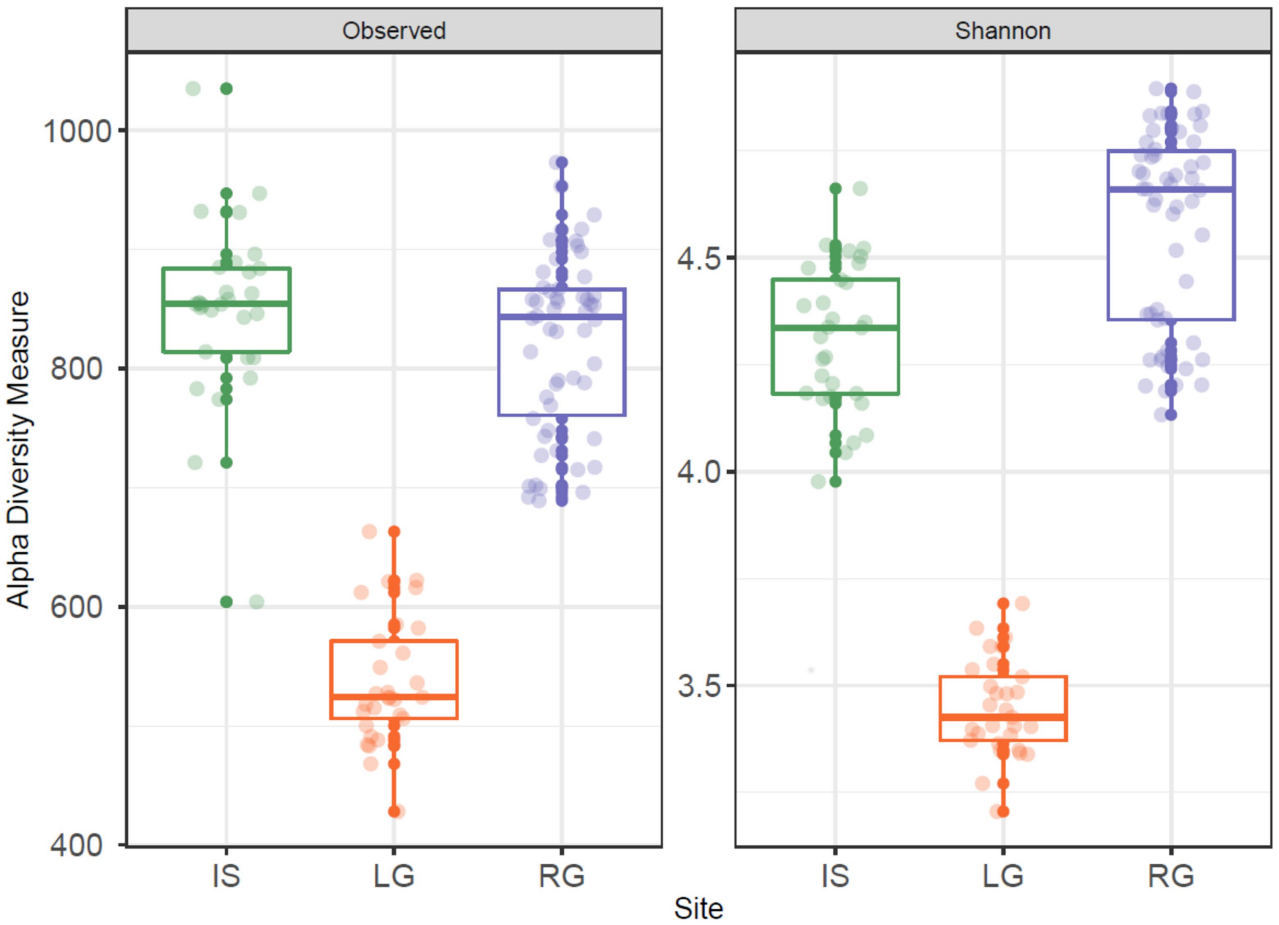
Observed number of OTUs (left panel) and Shannon diversity (right panel) for the three proglacial streams. In each panel, Isunnguata Sermia (IS) is to the left in green, Leverett (LG) in the middle in orange, and Russell (RG) to the right in purple.

In terms of alpha diversity, both the number of OTUs and Shannon diversity were higher in IS (Richness: mean = 849.3, range 604–1,035; Shannon: mean = 4.31, range 3.98–4.66) and RG (Richness: mean = 821.6, range 689–973; Shannon: mean = 4.57, range 4.13–4.9) when compared to LG (Richness: mean = 536.8, range 428–663; Shannon: mean = 3.45, range 3.21–3.69, Figure 3). Sampling site was a significant factor for both observed OTU richness (ANOVA, *F* = 191.4, *p* < 0.001) and Shannon diversity (ANOVA, *F* = 326.6, *p* < 0.001), and pair-wise comparisons between the catchments were also significant when each site was compared to each other (TukeyHSD, *p* < 0.01 for all comparisons). These differences in diversity were likely a function of the streams’ interaction (or lack thereof) with the non-glaciated catchment. Specifically, IS and RG were sampled further downstream from their glacial termini than LG, which was sampled close to the subglacial portal. As a result, RG and IS had greater proportions of proglacial (rather than glacierized) catchment cover (Table 1), and therefore a greater diversity of cells would be expected to originate from the adjacent terrestrial environment in these sites, compared to LG. The IS river in particular was broad and highly braided, and the continuous erosion of the lateral streambanks along with aerial deposition (e.g., from dust) may have further contributed to microbial diversity.

On the other hand, RG had several slow moving “pond” areas upstream of the sampling site, which likely had a strong influence on microbial assemblages (Crump et al., 2012; Ruiz-González et al., 2015; Comte et al., 2018). Upstream ponding may reduce stream velocity, allowing some of the glacial sediment to settle, thus potentially depositing a subset of the sediment-associated microbes. Proglacial freshwater habitats furthermore give rise to numerous ecological niches (Brandani et al., 2022), the microbial cells from which may be subsequently entrained and transported downstream. The RG and IS rivers serve as a contrast to the LG river, which by comparison has a streambank armored by bedrock and boulder scree, with little lateral erosion and no significant tributaries above the sampling site, and may help explain the lower observed diversity at this site.

**Figure 4:**
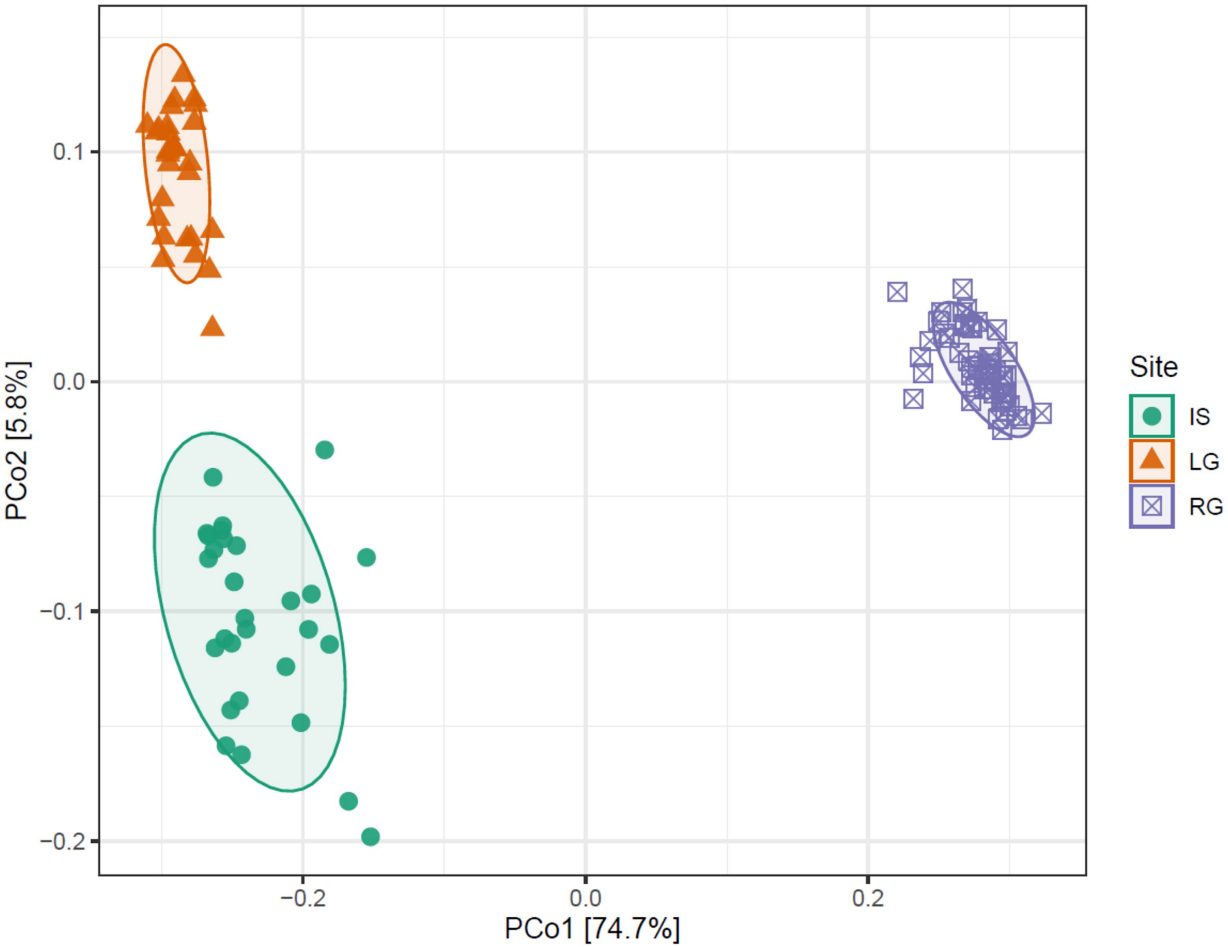
Principal coordinates analysis showing the difference between three GrIS sites on Bray-Curtis distances. Isunnguata Sermia (IS) in green, Leverett (LG) in orange, and Russell (RG) in purple.

However, it was not only the alpha diversity that differed between the three catchments, but also the assemblage composition. When Bray-Curtis distances were compared in a PCoA, the first two axes explained 80.5% of variability (the first axis explained 74.7%, and the second axis explained 5.8%). IS and LG clustered closely together along axis 1, while RG clustered away from the former two (Figure 4). These differences in assemblage structure between sites were significant when tested with PERMANOVA (*R*^2^ = 0.79, *F* = 211.33, *p* < 0.001). As discussed previously, RG likely clusters apart due to the freshwater habitats in its proglacial catchment coupled with its relatively low glaciated catchment cover. This contrasts with LG, which had very little deglaciated catchment cover, which likely limited proglacial inputs. However, it is interesting that IS does not cluster with RG, but rather with LG. While IS certainly has a large proglacial catchment, the glacierized catchment in IS was the biggest of the three, and the majority of the water in the river comes from the GrIS, even though there are lateral inputs *en route*. IS river is also massive compared to RG, and so lateral inputs might have smaller effects (i.e., are diluted) compared to a river the size of RG’s. Indeed, the interaction between the IS glacier and its glacier bed might have also played an important role in assemblage composition here as well. There is evidence of a high increase of glacier thickness in a relatively small area of the glacier snout of IS (Lindbäck et al., 2014), which may increase glacio-eustatic pressure to the glacier bed leading to subsoil deepening. The water is then pushed through sediment and leaves the glacier in the form of upwelling. Which may have been especially influential at the beginning of the flow season (i.e., the period sampled here), before meltwater from other sources became influential.

In total, 8,172 OTUs were identified in the full rarefied dataset. The five most abundant OTUs across the whole dataset (from highest to lowest) were *Rhodoferax* sp., *Flavobacterium* sp., *Methylobacter* sp., *Polaromonas* sp. and *Methylophilus* sp. *Rhodoferax* sp. was also the most abundant OTU when looking at each site separately (Supplementary Figure S1). Despite these similarities in the most abundant taxa, it is also apparent on the heatmap that sites differed compositionally, with the composition of IS and LG being more similar to that of RG, reflecting the results from the PCoA. A LefSe (Linear discriminant analysis Effect Size, Supplementary Figure S2) analysis was conducted to identify taxonomic units most likely to be characteristic to each, and to statistically explore differences in taxonomic composition between catchments. Results from the LEfSe indicate that IS differed by having a greater contribution of *Actinobacteria*, a common group in soils (Hazarika and Thakur, 2020). The origin of these soil bacteria in the IS proglacial stream might therefore be a mixture of the two processes described above, namely lateral erosion and dust deposition. On the other hand, we cannot dispute potential subglacial sources of soil bacteria, mainly by glacial erosion of overridden mature soils (Vinšová et al., 2022).

RG on the other hand was disproportionately represented by methylotrophs such as *Methylococcales*. The origin of this group in RG is unclear, but might be found in the lakes and ponds preceding the sampling site which likely contribute cells/DNA to the stream, since this group is also found in arctic tundra lakes (Somers et al., 2020). However, another conspicuous source may be from the subglacial environment, as several studies performed in the marginal area of RG have observed methylotrophs and/or elevated concentration of methane escaping the GrIS (Dieser et al., 2014; Christiansen and Jørgensen, 2018; Christiansen et al., 2021; Pain et al., 2021). Meanwhile, LG showed a significant proportion of taxa such as *Rhodoferax* and *Gallionellaceae*, with *Rhodoferax* being a typical cryospheric taxon (Bourquin et al., 2022), and are almost certainly indicators of the glacial environment, and likely of subglacial origin (Vinšová et al., 2022).

### Temporal trends

**Figure 5:**
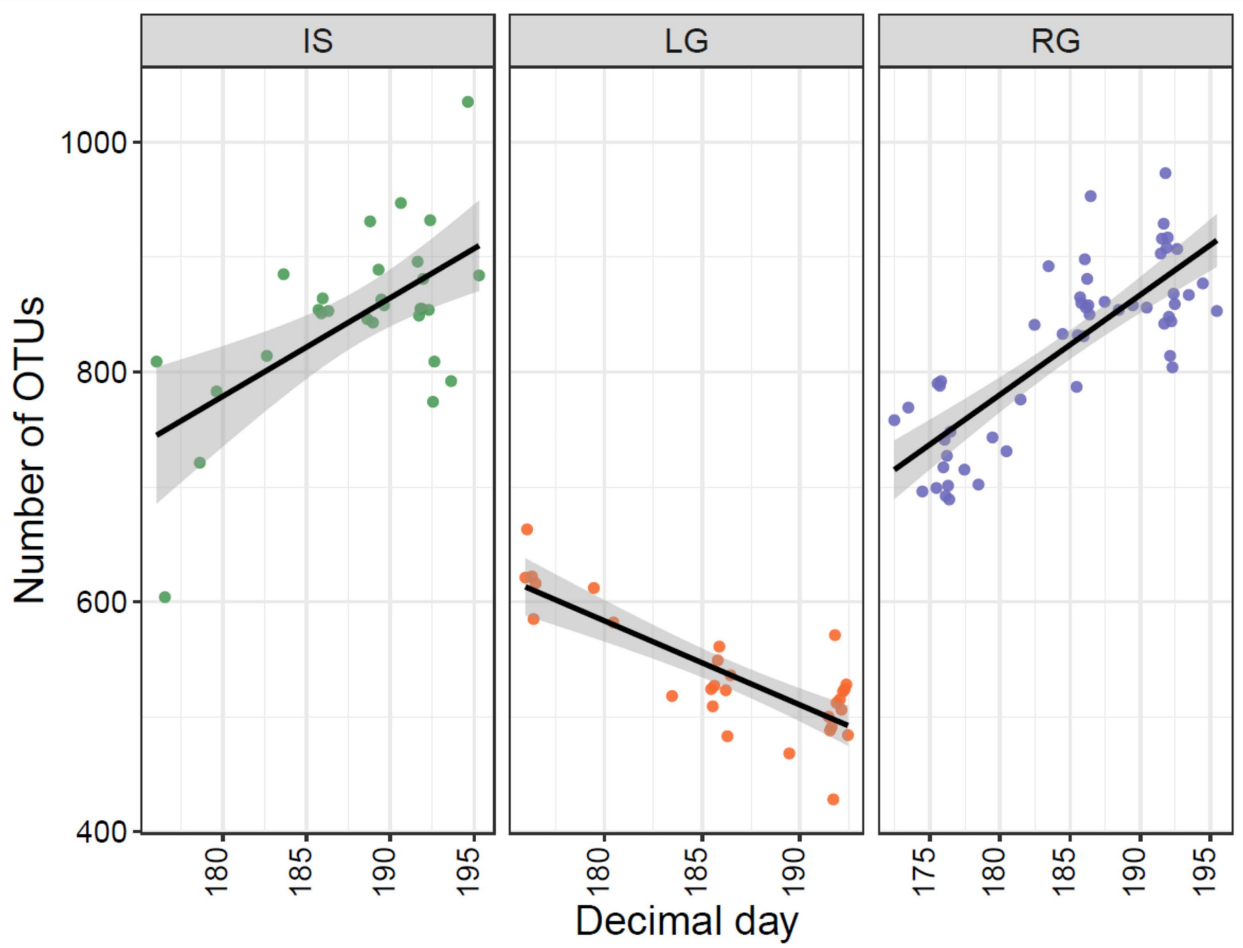
Observed number of OTUs plotted against Julian day (2018) samples were taken from the three GrIS proglacial streams, with Isunnguata Sermia (IS) to the left, Leverett (LG) in the middle, and Russell (RG) to the right.

Microbial assemblages changed over the monitored period as the hydrologically active glacial catchment size expanded and as the hydrological connection with the proglacial environment likely developed. Interestingly, microbial richness (i.e., the number of OTUs) in IS and RG increased over the sampling period, while LG richness decreased (*p* < 0.001 for all sites, Figure 5). For IS and RG, the increase in richness may be attributed to greater hydrological connectivity within the proglacial catchment over time. Adjacent freshwater ecosystems become hydrologically activated with snowmelt, and likely undergo succession throughout the summer, bearing influence on the bacterial assemblages that they contribute to the streams (Brandani et al., 2022).

The declining richness for LG over the summer is more difficult to explain. We hypothesize the reason for this is likely related to changes taking place within the glacierized catchment (i.e., rather than the non-glaciated catchment) given the limited interaction of this river with the proglacial landscape above the sampling site. Specifically, given the well-known evolution of an inefficient distributed hydrological system to an efficient subglacial drainage system at LG (Chandler et al., 2013; Lamarche-Gagnon et al., 2019), the heterogeneity of possible cell sources might be reduced with the formation of a channelized subglacial drainage system (Dubnick et al., 2017). In other words, while other sites are presumably adding diversity through greater interaction with the adjacent proglacial catchment, at LG richness was reduced as subglacial hydrological efficiency improved, which homogenized the potential source pool of microbes downstream.

Environmental correlations for individual OTUs were substantially different between the three catchments (Supplementary Figure S3). No OTUs had a significant association with discharge in RG (which showed little change in discharge over the study period), and genera which typically occur in the cryosphere, including *Rhodoferax*, *Polaromonas*, and *Methylotenera* (Bourquin et al., 2022) decreased over the sampling period, indicating an increasing non-cryospheric component to the overall assemblage. Meanwhile, relative abundances of the cryospheric bacterium *Polaromonas* increased over the summer in LG. Soil bacteria such as *Luteolibacter* increased over the summer in all sites, but especially at IS and LG. Furthermore, the anaerobic digester *Marinilabiliales* sp. decreased in IS and LG, but increased in RG (Supplementary Figure S3).

LG had the most significant associations with individual OTUs and hydrochemical variables, possibly due to streamwater originating disproportionately from one source (Supplementary Figure S3). OTUs were significantly correlated with pH and conductivity at LG, probably due to these variables being related to changes in subglacial drainage flowpaths, and subglacial drainage being a more important consideration at LG than at other sites. IS was an intermediate site, with none of our measured hydrological or chemical variables having consistent influence over the relative abundance of the chosen OTUs.

That said, many of the OTUs themselves seemed influenced by environmental variables. These included methylotrophs such as *Methylophilus, Methylobacter, Methylotenera* and *Methylococcales sp*. All of these microbes had decreasing trends with DOY in the case of IS and LG, whereas in RG some (i.e., *Methylophilus* and *Methylococcales sp.*), were increasing over the study period. Recent studies have been performed focusing on methane export in all of these sites (IS − Christiansen et al., 2021; LG − Lamarche-Gagnon et al., 2019; RG − Dieser et al., 2014), but it seems that RG stands out from the rest as a methane export hotspot and by its abundance of methylotrophs (Dieser et al., 2014; Christiansen and Jørgensen, 2018; Christiansen et al., 2021). In accordance with other studies (Stibal et al., 2012b; Lawson et al., 2014; Kohler et al., 2017), the carbon content in RG suspended sediments (mean 0.93 ± 0.34% standard deviation; range 0.34–1.32%) was much higher than at LG (mean 0.09 ± 0.02% standard deviation; range 0.06–0.12%) but also than at IS (mean 0.04 ± 0.02% standard deviation; range 0.02–0.07%), which might also explain this higher amount of methylotrophs compared to the other two sites.

Lastly, no detectable changes were observed in richness nor community composition over diel timescales (i.e., the “daily” samples). While this could of course be due to our relatively low number of samples/replicates, as well as the early period of the melt season from which the samples were taken (i.e., when diurnal peaks in discharge are less pronounced than later in the season), we tentatively hypothesize that the seasonal variability associated with the melt season is much more important than changes that occur over daily timescales.

### Patterns in putative metabolisms

In order to further investigate how the putative functionality of microbial assemblages changed over the melt season, we selected the main metabolism types of the most abundant OTUs (reaching at least 0.99% abundance in at least 1 sample) in our dataset for further analysis (Supplementary Table S2). A considerable caveat applies to these analyses, in that bacterial functions were designated by database matches, rather than through functional assays or DNA- or RNA-based metagenomic or metatranscriptomic sequencing. Furthermore, results are also prone to compositionality that results from working with proportional data, although efforts to minimize this were taken (i.e., calculating relative abundances from the selected OTUs rather than the full dataset).

**Figure 6:**
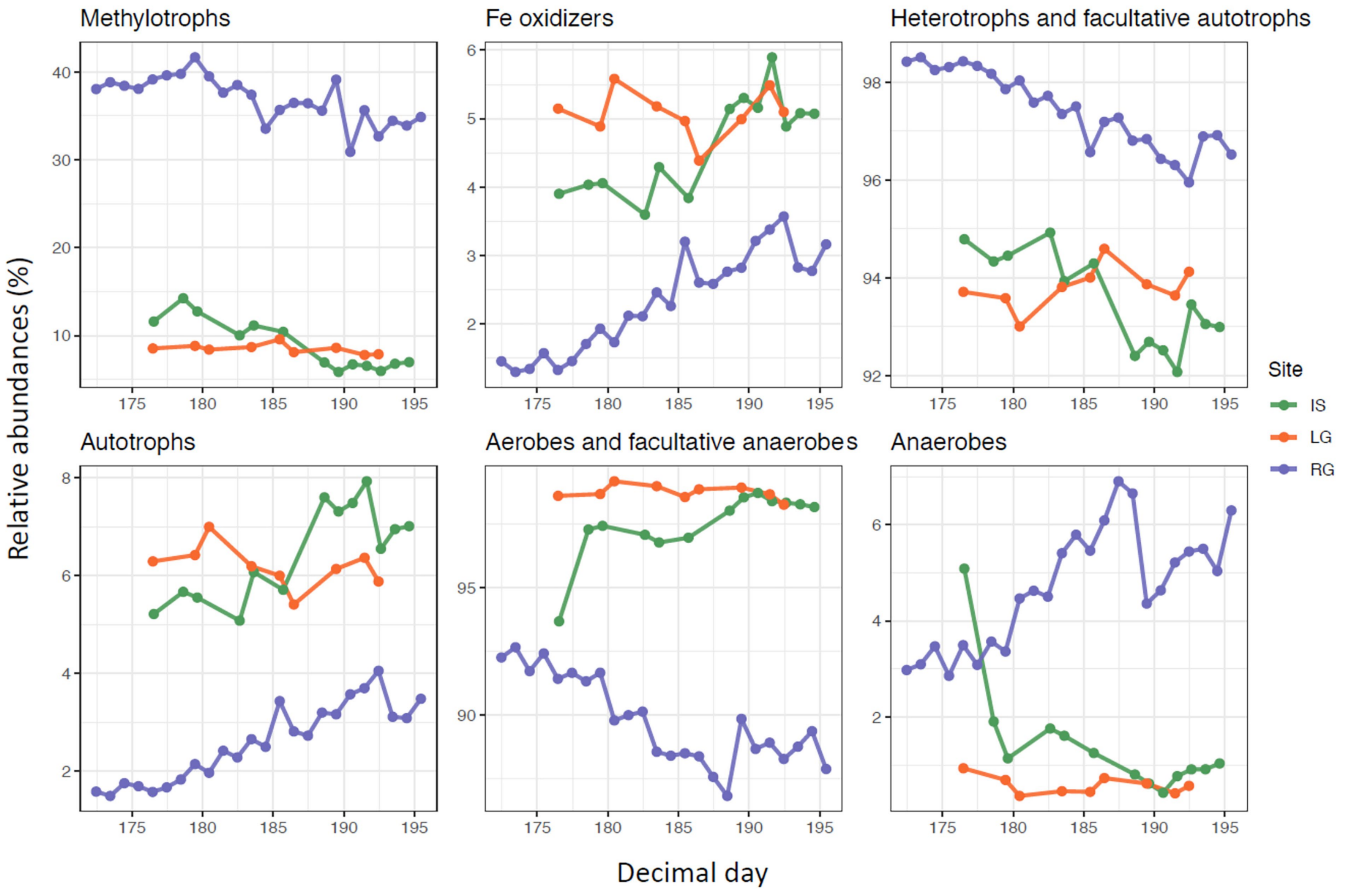
Relative abundance of OTUs for selected metabolism types plotted against Julian day (2018). Please note the different y-axis scales.

Most of the relative abundance of all sites were captured within “aerobes and facultative anaerobes” and “heterotrophs/facultative autotrophs.” Heterotrophs and facultative autotrophs exceeded the number of “autotrophs” at all sites. For RG and IS, heterotrophs and facultative autotrophs decreased in relative abundance over the course of the study (*p* < 0.001 for both, Figure 6). In RG and IS, we furthermore observed a decreasing trend of aerobes and facultative anaerobes (*p* < 0.001 and *p* = 0.002, respectively) and an increase of anaerobes during the observational period (*p* < 0.001 for both, Figure 6). However, these groups remained relatively stable over time in the case of LG, although the first sample for IS differed from the rest. Potentially, the higher proportion of anaerobes (and similarly the proportion of aerobes) in IS earlier in the monitoring period might point to the evolution of the hydrological catchment within the glacial catchment, indicating a greater proportion of subglacial organisms at this time.

At the same time, “autotroph” relative abundances increased in RG and IS (both *p* < 0.001), but not LG. This increase in autotroph relative abundance over time might indicate both an increase in connectivity with non-glacial tributaries which may be more likely to harbor such taxa (Brandani et al., 2022), but also growth and successional patterns within communities inhabiting proglacial areas in general (Kong et al., 2019). Alternatively, it might also be caused by the growing connection between the supra- and subglacial systems and the higher input of the surface autotrophs specifically to the proglacial stream (Dubnick et al., 2017).

Methylotrophs (as a subgroup of heterotrophs) constituted between 30 and 40% of the relative abundances calculated within the top OTUs at RG, though only about 5–10% for IS and LG. This comparatively high abundance of methylotrophs at RG further suggests that special conditions occur here which are suitable for this particular metabolism type (Christiansen et al., 2021) in comparison to the other two systems. There was a decreasing trend of methylotrophs for both RG and IS over time (*p* < 0.001 for both, Figure 6), but again not for LG. Lastly, Fe oxidizers represented a minority of relative abundances at all sites, and were proportionately in greater abundance in both IS and LG compared to RG (Figure 6). While both RG and IS showed an increase in the relative abundance of Fe oxidizers with DOY (*p* < 0.001 for both), there was no trend in LG.

Collectively, the lack of temporal trends in metabolism types at LG potentially indicates both a lack of connectivity with the surrounding proglacial catchment area, but also a relatively consistent range of habitats being drained by this river over the course of the study period. Overall, the limited effect of seasonality among the different metabolism types observed from these catchments might be due to the origin of the microbes collected from the water column. Since microbes are primarily flushed from upstream environments, temporal patterns in this study are more reflective of seasonal shifts taking place in these upstream habitats than within the microbial communities growing directly at the sampling site. In addition, the seasonal changes may reflect evolution of the drainage topology and advancing sediment erosion rather than temporal changes of microbial communities. With this in mind, greater metabolic shifts might be observed if sampling was conducted over a wider temporal interval. For example, microbes with much different metabolisms might make up a larger proportion of the assemblage if the sampling were to capture the onset of glacier melt, which would coincide with the drainage of potentially much different habitat types.

## Conclusion

We investigated how catchment-level differences influence the structure of suspended microbial assemblages recovered from three rivers draining the GrIS through monitoring. Our results suggest that both glacial and proglacial hydrological processes are likely influential to some degree for the resulting assemblage structure. Specifically, the ratio of glacierized to proglacial catchment coverage and the hydrological connectivity with different habitat types seems to play a bigger role in assemblage structure than the absolute catchment size or discharge. Importantly, the interaction of stream water with corresponding non-glaciated proglacial catchments likely evolve over the course of the summer, leading to substantial changes to assemblage structure over the melt season. Although we argue that our interpretations for the observed patterns make ecological sense, it will be necessary to validate these interpretations with new field efforts to definitively isolate mechanisms. Specifically, further efforts to characterize and quantify microbial contributions from the proglacial watershed is much needed, as are quantitative metabolic profiling of microbial communities derived from these and other cold freshwater environments. Nonetheless, these results are important for hydrologists and biogeochemists using microbial cells as indicators for hydrological flow paths, which are likely to change in these sensitive landscapes with climate warming.

## Data availability statement

The datasets presented in this study can be found in online repositories. The names of the repository/repositories and accession number(s) can be found at: https://www.ncbi.nlm.nih.gov/genbank/, OP279921-OP279953.

## Author contributions

MSt, TK, JH, and JW conceived of the project. TK, PV, MB, GL-G, AH, JH, AK, SH, KC, and MSt collected the samples. KV, TK, PV, LF, and GL-G performed the labwork. KV, LF, MSc, and TK performed the analyses. KV, TK, and MSt wrote the paper along with significant input and editing from all coauthors. All authors contributed to the article and approved the submitted version.

## Funding

This research was supported by Czech Science Foundation (18-12630S) and the Czech Ministry of Education, Youth, and Sport (ERC CZ LL2004) grants to MS. Greenland fieldwork was additionally supported by a Leverhulme Trust Research Grant (RPG-2016-439) to JLW. TJK was supported by the Charles University project PRIMUS/22/SCI/001 and by the Charles University Research Centre program no. 204069. ADH was supported by Monica Cole and Gino Watkins Memorial grants from the Royal Geographical Society and the Scott Polar Institute, respectively.

## Conflict of interest

The authors declare that the research was conducted in the absence of any commercial or financial relationships that could be construed as a potential conflict of interest.

## Publisher’s note

All claims expressed in this article are solely those of the authors and do not necessarily represent those of their affiliated organizations, or those of the publisher, the editors and the reviewers. Any product that may be evaluated in this article, or claim that may be made by its manufacturer, is not guaranteed or endorsed by the publisher.
