# Supplementary material for: Catchment characteristics and seasonality control the composition of microbial assemblages exported from three outlet glaciers of the Greenland Ice Sheet
Source: Front Microbiol. 2022 Nov 29;13:1035197. doi: 10.3389/fmicb.2022.1035197 (PMC9745319; doi:10.3389/fmicb.2022.1035197)
Supplement: Supplementary file 1 [file Data_Sheet_1.docx]

**
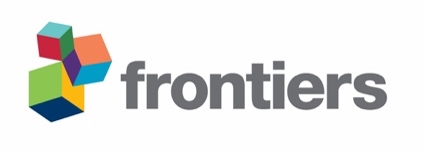
**

Supplementary Material

# Supplementary Figures


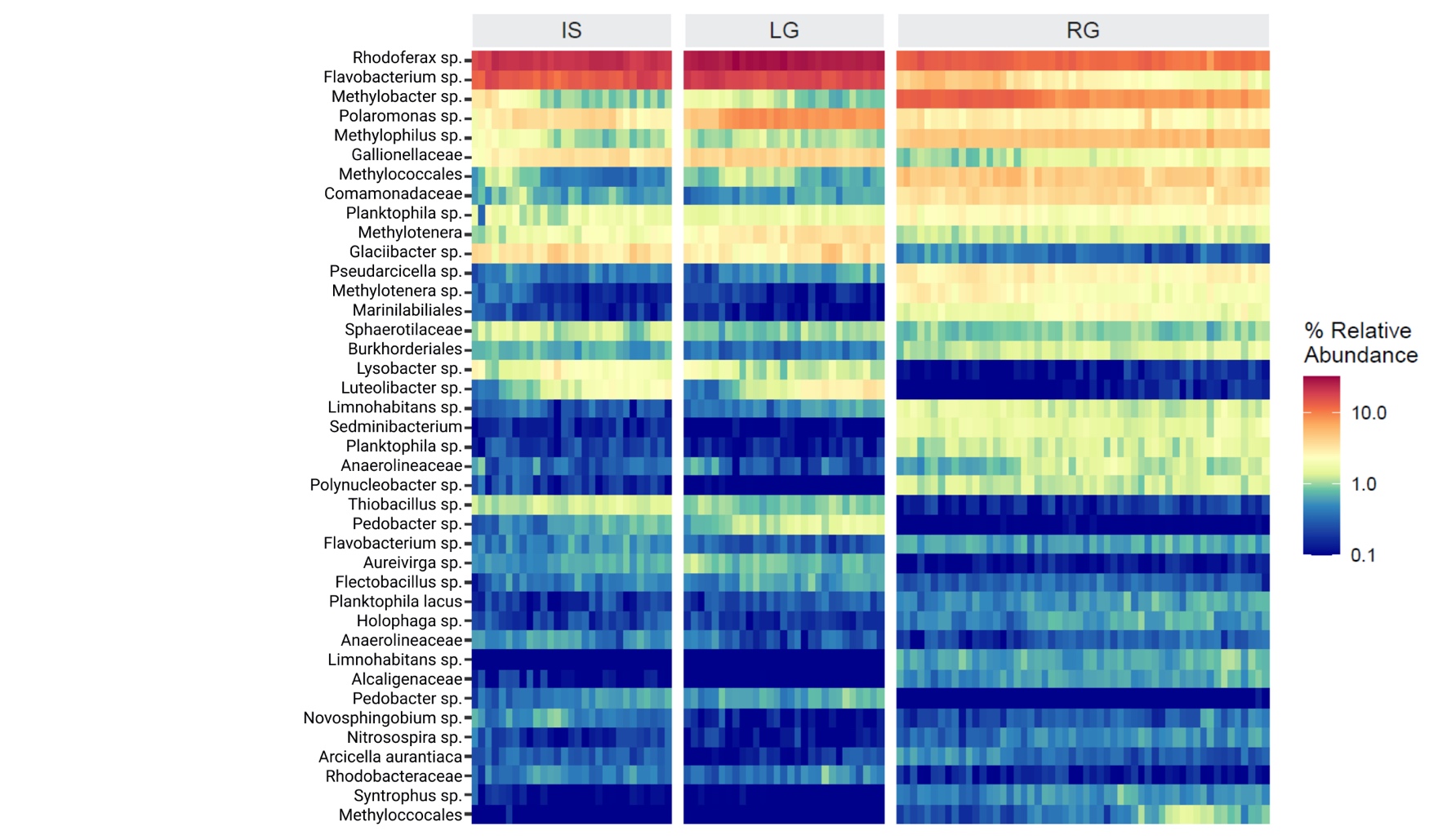


**Figure S1**: Heatmap of the top 40 taxa by relative abundance plotted for each sample, ordered by day sampled (from the beginning to the end) and separated by GrIS catchment.


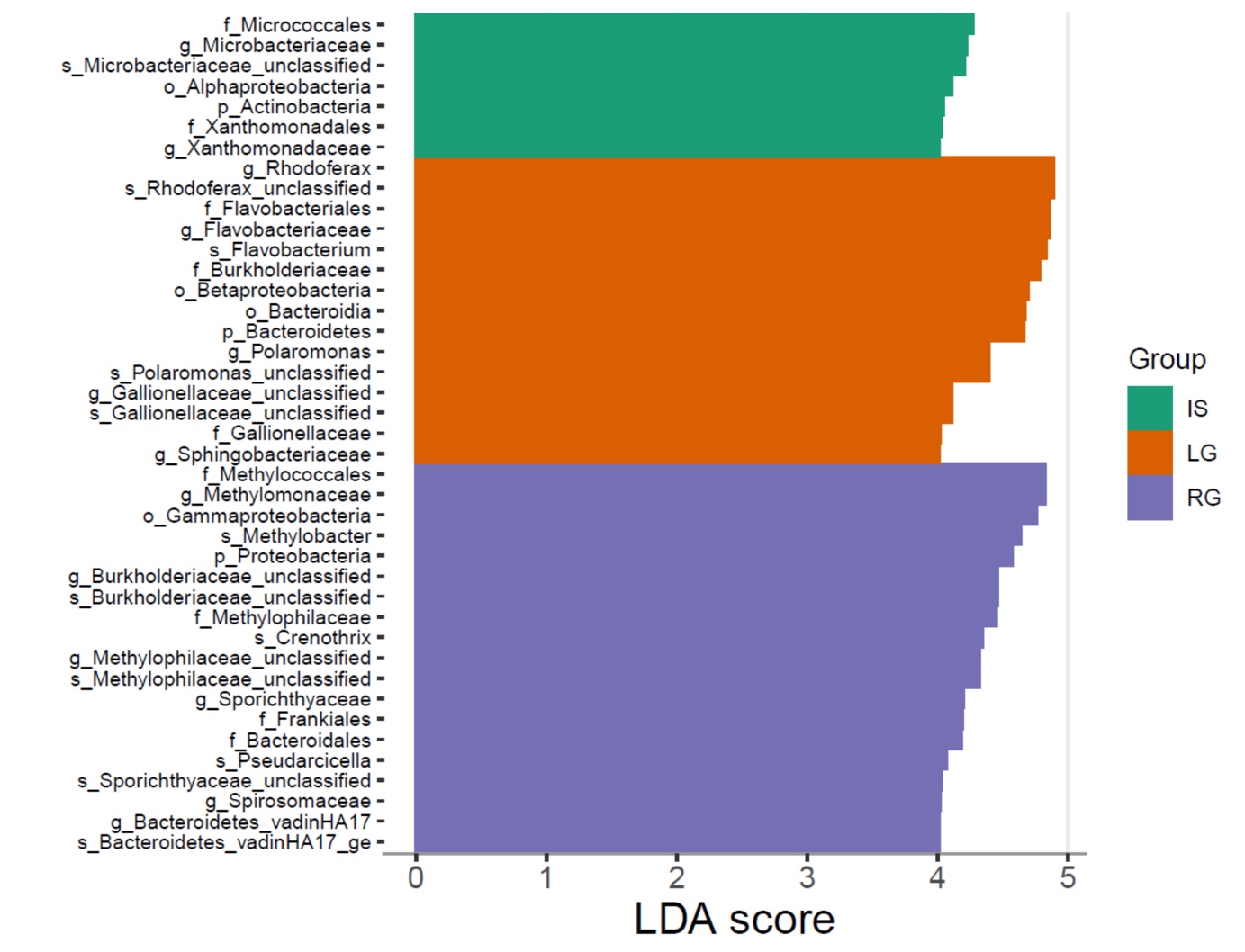


**Figure S2**: LEfSe (Linear discriminant analysis Effect Size) results showing the taxonomic features most likely to explain differences between the three sampled GrIS catchments. Please note that higher taxonomic levels reflect the high abundance of a subset of organisms at lower taxonomic levels. In total, 13 out of 41 rows originated by repeating the same organisms at a higher taxonomic level. The Silva database is used for naming groups.

**
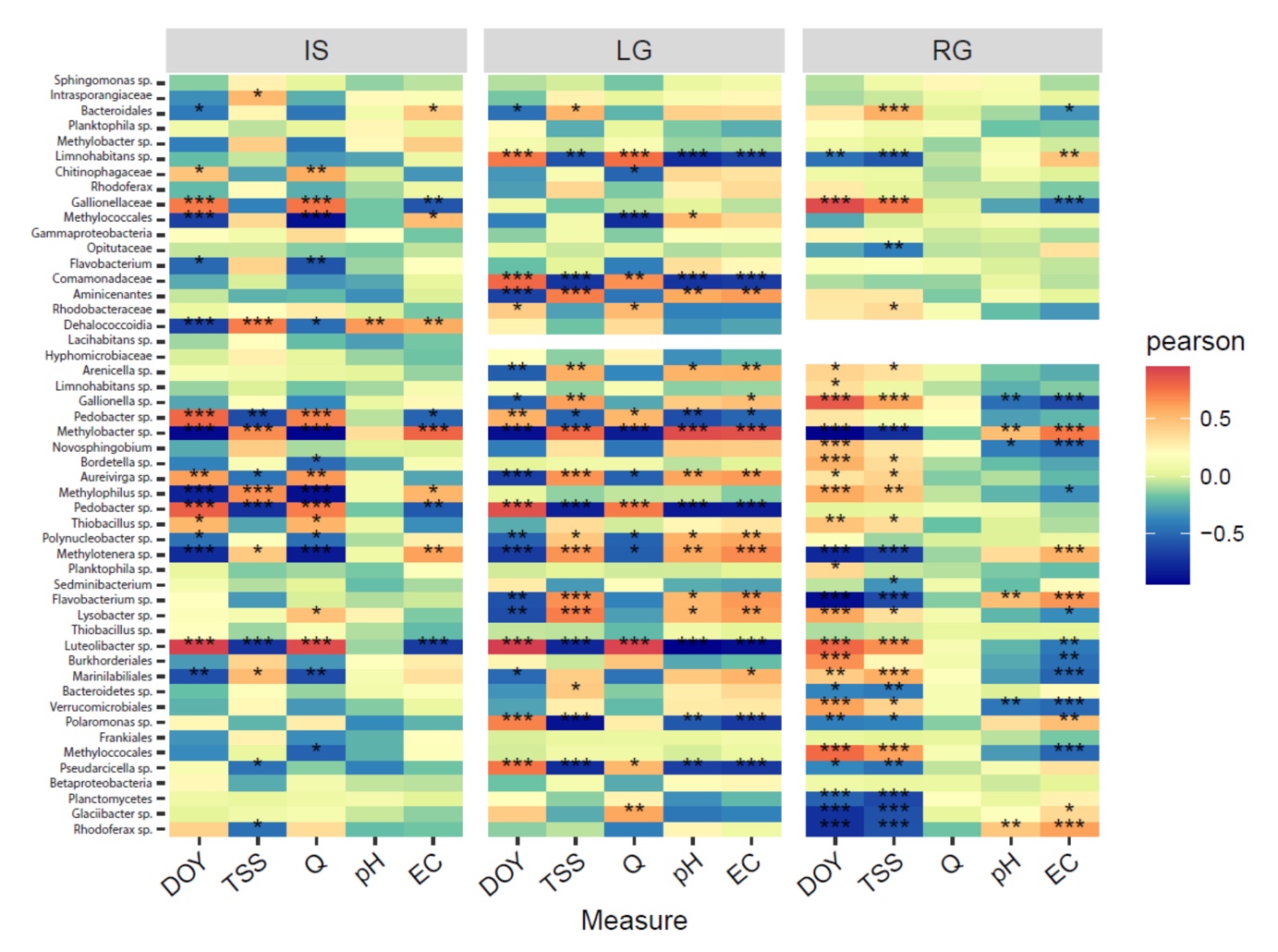
**

**Figure S3**: Correlation heatmap of differentially abundant taxa obtained by random forest analysis compared with the monitored variables for each GrIS catchment.

# Supplementary Tables

**Table S1**: List of samples with relevant metadata.

| **Site** | **Day** | **Decimal day** | **pH** | **TSS**  **(g L^-1^)** | **EC**  **(µS cm^-1^)** | **Water temp (°C)** | **Discharge (m^3^ s^-1^)** |
| --- | --- | --- | --- | --- | --- | --- | --- |
| IS | 25 June 2018 | 176.042 | 7.08 | 4.92 | 29.20 | 3.44 | 134.12 |
| IS | 25 June 2018 | 176.542 | 6.98 | 4.98 | 27.03 | 2.67 | 129.35 |
| IS | 27 June 2018 | 178.625 | 6.92 | 4.24 | 29.16 | 7.17 | 103.86 |
| IS | 28 June 2018 | 179.625 | 6.94 | 3.25 | 29.43 | 6.44 | 122.47 |
| IS | 1 July 2018 | 182.625 | 6.83 | 2.71 | 26.30 | 4.70 | 117.73 |
| IS | 2 July 2018 | 183.625 | 6.77 | 1.84 | 22.50 | 6.06 | 118.83 |
| IS | 4 July 2018 | 185.708 | 6.75 | 1.76 | 20.55 | 6.87 | 160.78 |
| IS | 4 July 2018 | 185.875 | 6.80 | 2.16 | 21.34 | 6.87 | 172.08 |
| IS | 4 July 2018 | 185.958 | 6.82 | 2.28 | 22.03 | 6.87 | 175.87 |
| IS | 5 July 2018 | 186.292 | 6.84 | 3.63 | 21.49 | 6.87 | 172.57 |
| IS | 7 July 2018 | 188.625 | 6.77 | 2.44 | 20.06 | 6.87 | 256.67 |
| IS | 7 July 2018 | 188.792 | 6.84 | 2.28 | 20.69 | 6.87 | 280.52 |
| IS | 7 July 2018 | 188.958 | 6.90 | 3.02 | 21.74 | 6.87 | 296.98 |
| IS | 8 July 2018 | 189.292 | 6.83 | 2.85 | 20.99 | 6.87 | 282.12 |
| IS | 8 July 2018 | 189.458 | 6.83 | 2.97 | 20.51 | 6.87 | 276.58 |
| IS | 8 July 2018 | 189.625 | 6.81 | 2.12 | 21.22 | 6.87 | 278.68 |
| IS | 9 July 2018 | 190.625 | 6.90 | 2.23 | 23.09 | 6.87 | 308.29 |
| IS | 10 July 2018 | 191.625 | 6.87 | 1.66 | 21.99 | 6.87 | 307.99 |
| IS | 10 July 2018 | 191.708 | 6.87 | 1.76 | 22.35 | 6.87 | 306.83 |
| IS | 10 July 2018 | 191.792 | 6.90 | 1.75 | 22.56 | 6.87 | 316.43 |
| IS | 10 July 2018 | 191.875 | 6.91 | 1.91 | 22.55 | 6.87 | 334.88 |
| IS | 10 July 2018 | 191.958 | 6.94 | 2.15 | 22.66 | 6.87 | 352.32 |
| IS | 11 July 2018 | 192.292 | 6.86 | 1.55 | 21.28 | 6.87 | 325.04 |
| IS | 11 July 2018 | 192.375 | 6.85 | 1.49 | 20.99 | 6.87 | 314.20 |
| IS | 11 July 2018 | 192.542 | 6.85 | 1.69 | 20.91 | 6.87 | 278.99 |
| IS | 11 July 2018 | 192.625 | 6.86 | 1.83 | 21.30 | 6.87 | 273.59 |
| IS | 12 July 2018 | 193.625 | 6.87 | 1.49 | 19.76 | 6.87 | 286.63 |
| IS | 13 July 2018 | 194.625 | 6.85 | 1.55 | 19.75 | 6.87 | 314.12 |
| IS | 14 July 2018 | 195.292 | 6.85 | 2.15 | 17.48 | 6.87 | 279.18 |
| LG | 24 June 2018 | 175.958 | 7.41 | 1.25 | 21.16 | 6.87 | 107.20 |
| LG | 25 June 2018 | 176.042 | 7.41 | 1.23 | 21.04 | 6.87 | 103.29 |
| LG | 25 June 2018 | 176.292 | 7.44 | 1.21 | 22.29 | 6.87 | 97.30 |
| LG | 25 June 2018 | 176.375 | 7.44 | 1.21 | 22.97 | 6.87 | 91.79 |
| LG | 25 June 2018 | 176.476 | 7.45 | 1.21 | 23.37 | 6.87 | 92.60 |
| LG | 28 June 2018 | 179.458 | 7.39 | 0.97 | 22.33 | 6.87 | 76.00 |
| LG | 29 June 2018 | 180.458 | 7.35 | 0.98 | 19.49 | 6.87 | 80.94 |
| LG | 2 July 2018 | 183.458 | 7.18 | 0.90 | 14.43 | 6.87 | 89.14 |
| LG | 4 July 2018 | 185.458 | 7.11 | 0.86 | 13.18 | 6.87 | 101.73 |
| LG | 4 July 2018 | 185.542 | 7.11 | 0.86 | 13.17 | 6.87 | 102.31 |
| LG | 4 July 2018 | 185.625 | 7.10 | 0.86 | 12.84 | 6.87 | 106.37 |
| LG | 4 July 2018 | 185.795 | 7.03 | 0.88 | 11.52 | 6.87 | 109.82 |
| LG | 4 July 2018 | 185.879 | 7.00 | 0.88 | 10.92 | 6.87 | 108.02 |
| LG | 5 July 2018 | 186.208 | 7.03 | 0.86 | 11.23 | 6.87 | 101.77 |
| LG | 5 July 2018 | 186.292 | 7.05 | 0.86 | 11.44 | 6.87 | 103.07 |
| LG | 5 July 2018 | 186.458 | 7.07 | 0.87 | 11.85 | 6.87 | 98.67 |
| LG | 8 July 2018 | 189.458 | 6.96 | 0.86 | 9.68 | 6.87 | 149.62 |
| LG | 10 July 2018 | 191.469 | 6.77 | 0.83 | 9.71 | 6.87 | 173.55 |
| LG | 10 July 2018 | 191.542 | 6.59 | 0.83 | 9.80 | 6.87 | 179.29 |
| LG | 10 July 2018 | 191.625 | 6.76 | 0.83 | 9.64 | 6.87 | 188.27 |
| LG | 10 July 2018 | 191.708 | 6.73 | 0.84 | 9.15 | 6.87 | 194.18 |
| LG | 10 July 2018 | 191.792 | 6.70 | 0.83 | 8.71 | 6.87 | 203.42 |
| LG | 10 July 2018 | 191.875 | 6.68 | 0.82 | 8.39 | 6.87 | 206.96 |
| LG | 11 July 2018 | 192.042 | 6.47 | 0.82 | 8.15 | 6.87 | 200.64 |
| LG | 11 July 2018 | 192.125 | 6.68 | 0.81 | 8.37 | 6.87 | 190.04 |
| LG | 11 July 2018 | 192.208 | 6.71 | 0.81 | 8.69 | 6.87 | 186.26 |
| LG | 11 July 2018 | 192.292 | 6.73 | 0.81 | 9.01 | 6.87 | 179.48 |
| LG | 11 July 2018 | 192.375 | 6.76 | 0.82 | 9.34 | 6.87 | 183.95 |
| LG | 11 July 2018 | 192.458 | 6.77 | 0.82 | 9.61 | 6.87 | 183.22 |
| RG | 21 June 2021 | 172.458 | 6.99 | 0.45 | 13.03 | 6.87 | 33.68 |
| RG | 22 June 2021 | 173.458 | 7.00 | 0.45 | 12.32 | 6.87 | 33.29 |
| RG | 23 June 2021 | 174.458 | 6.98 | 0.45 | 12.08 | 6.87 | 29.60 |
| RG | 24 June 2021 | 175.458 | 7.00 | 0.46 | 11.68 | 6.87 | 25.16 |
| RG | 24 June 2021 | 175.542 | 6.98 | 0.46 | 11.41 | 6.87 | 27.21 |
| RG | 24 June 2021 | 175.708 | 6.95 | 0.47 | 10.89 | 6.87 | 31.39 |
| RG | 24 June 2021 | 175.792 | 6.94 | 0.46 | 10.78 | 6.87 | 30.90 |
| RG | 24 June 2021 | 175.958 | 6.96 | 0.45 | 11.41 | 6.87 | 33.67 |
| RG | 25 June 2021 | 176.042 | 6.98 | 0.45 | 11.76 | 6.87 | 30.77 |
| RG | 25 June 2021 | 176.125 | 7.00 | 0.44 | 11.94 | 6.87 | 30.12 |
| RG | 25 June 2021 | 176.208 | 7.00 | 0.44 | 12.05 | 6.87 | 28.45 |
| RG | 25 June 2021 | 176.292 | 6.99 | 0.44 | 12.08 | 6.87 | 27.52 |
| RG | 25 June 2021 | 176.375 | 6.99 | 0.45 | 12.03 | 6.87 | 26.86 |
| RG | 25 June 2021 | 176.458 | 6.99 | 0.45 | 11.87 | 6.87 | 25.98 |
| RG | 26 June 2021 | 177.458 | 7.06 | 0.52 | 12.30 | 6.87 | 21.37 |
| RG | 27 June 2021 | 178.458 | 7.05 | 0.47 | 12.52 | 6.87 | 23.74 |
| RG | 28 June 2021 | 179.458 | 6.99 | 0.46 | 11.20 | 6.87 | 27.41 |
| RG | 29 June 2021 | 180.458 | 6.95 | 0.46 | 10.90 | 6.87 | 30.89 |
| RG | 30 June 2021 | 181.458 | 6.99 | 0.51 | 11.13 | 6.87 | 20.27 |
| RG | 01 July 2018 | 182.458 | 6.97 | 0.49 | 11.24 | 6.87 | 25.59 |
| RG | 2 July 2018 | 183.458 | 6.93 | 0.52 | 10.58 | 6.87 | 24.37 |
| RG | 3 July 2018 | 184.458 | 6.93 | 0.54 | 10.60 | 6.87 | 24.80 |
| RG | 4 July 2018 | 185.458 | 6.93 | 0.52 | 10.22 | 6.87 | 26.42 |
| RG | 4 July 2018 | 185.542 | 6.92 | 0.54 | 9.86 | 6.87 | 26.43 |
| RG | 4 July 2018 | 185.708 | 7.16 | 0.56 | 9.34 | 6.87 | 33.04 |
| RG | 4 July 2018 | 185.792 | 6.86 | 0.51 | 9.24 | 6.87 | 34.86 |
| RG | 4 July 2018 | 185.958 | 6.87 | 0.49 | 9.28 | 6.87 | 34.10 |
| RG | 5 July 2018 | 186.042 | 6.86 | 0.50 | 9.31 | 6.87 | 33.81 |
| RG | 5 July 2018 | 186.125 | 6.85 | 0.51 | 9.35 | 6.87 | 32.26 |
| RG | 5 July 2018 | 186.208 | 6.88 | 0.52 | 9.42 | 6.87 | 31.34 |
| RG | 5 July 2018 | 186.292 | 6.87 | 0.52 | 9.49 | 6.87 | 31.36 |
| RG | 5 July 2018 | 186.375 | 6.88 | 0.60 | 9.59 | 6.87 | 29.79 |
| RG | 5 July 2018 | 186.458 | 6.89 | 0.68 | 9.81 | 6.87 | 29.48 |
| RG | 6 July 2018 | 187.458 | 6.88 | 0.56 | 9.64 | 6.87 | 29.75 |
| RG | 7 July 2018 | 188.458 | 6.90 | 0.55 | 9.67 | 6.87 | 30.08 |
| RG | 8 July 2018 | 189.458 | 6.92 | 0.56 | 10.25 | 6.87 | 30.68 |
| RG | 9 July 2018 | 190.458 | 6.98 | 0.50 | 11.33 | 6.87 | 26.78 |
| RG | 10 July 2018 | 191.458 | 7.05 | 0.54 | 12.21 | 6.87 | 22.78 |
| RG | 10 July 2018 | 191.542 | 7.06 | 0.54 | 12.47 | 6.87 | 23.93 |
| RG | 10 July 2018 | 191.667 | 7.02 | 0.61 | 11.16 | 6.87 | 27.94 |
| RG | 10 July 2018 | 191.708 | 6.99 | 0.78 | 10.57 | 6.87 | 29.86 |
| RG | 10 July 2018 | 191.792 | 6.93 | 0.57 | 9.47 | 6.87 | 31.08 |
| RG | 10 July 2018 | 191.875 | 6.88 | 0.56 | 8.87 | 6.87 | 32.44 |
| RG | 10 July 2018 | 191.958 | 6.86 | 0.60 | 8.56 | 6.87 | 31.36 |
| RG | 11 July 2018 | 192.042 | 6.84 | 0.52 | 8.79 | 6.87 | 31.01 |
| RG | 11 July 2018 | 192.125 | 6.86 | 0.52 | 9.11 | 6.87 | 29.05 |
| RG | 11 July 2018 | 192.208 | 6.88 | 0.52 | 9.54 | 6.87 | 26.58 |
| RG | 11 July 2018 | 192.292 | 6.91 | 0.52 | 10.01 | 6.87 | 26.77 |
| RG | 11 July 2018 | 192.375 | 6.95 | 0.52 | 10.37 | 6.87 | 27.24 |
| RG | 11 July 2018 | 192.458 | 6.95 | 0.54 | 10.34 | 6.87 | 27.18 |
| RG | 11 July 2018 | 192.625 | 6.89 | 0.61 | 9.39 | 6.87 | 30.19 |
| RG | 12 July 2018 | 193.458 | 6.94 | 0.53 | 10.11 | 6.87 | 28.10 |
| RG | 13 July 2018 | 193.458 | 6.92 | 0.55 | 9.88 | 6.87 | 29.20 |
| RG | 14 July 2018 | 195.458 | 6.70 | 0.51 | NA | 6.87 | 37.30 |

|  |  | **1st significant BLAST hit** | **Table S2:** List of the most abundant OTUs and their putative functionality (GB=GenBank accession of a representative sequence of the OTU; id.=identity; cov.=coverage; Metabolism: Tr=trophy, H=heterotrophy, A=autotrophy, F=facultative autotrophy; O_2_: A=aerobic, N=anaerobic, F=facultatively anaerobic; N/S/H/Fe: R=reduce, O=oxidize; Pt=presence of phototrophy, F=facultative; Type: R=respiration, RM=respiration + methylotrophy, DHR=dehalorespiration). |  |  | **Metabolism type** | | | | | | | |  |
| --- | --- | --- | --- | --- | --- | --- | --- | --- | --- | --- | --- | --- | --- | --- |
| **OTU** | **GB** |  | **Identification** | **id. [%]** | **cov. [%]** | **Tr** | **O_2_** | **N** | **S** | **H** | **Fe** | **Pt** | **Type** | **Ecology of close BLAST hits, misc.** |
| 00001 | OP279921 | MT462144 | Rhodoferax sp. | 100 | 100 | H | F |  |  |  |  | F | R | spring, fish egg, lake, Fe-rich mat, river, salt marsh;  extremely common in water column and sediments |
| 00002 | OP279922 | MN519644 | Flavobacterium sp. | 100 | 100 | H | F | R |  |  |  |  | R | soil and freshwater |
| 00003 | OP279925 | LC378784 | Methylophilus sp. | 100 | 100 | H | A |  |  |  |  |  | RM | water, groundwater, drinkw., filter |
| 00004 | OP279923 | OK135604 | Methylobacter sp. | 100 | 100 | H | A |  |  |  |  |  | RM | lakes, sediment, arctic soil, under-ice methanotrophy;  cold methanotrophic habitats, water column, common |
| 00005 | OP279928 | MN477136 | Comamonadaceae | 98,4 | 100 | H |  |  |  |  |  |  | R | boreal lake, spring biofilm, freshwater sponge |
| 00006 | OP279926 | KR853535 | Methylococcales | 99,6 | 100 | H | A |  |  |  |  |  | RM | under-ice methanotrophy, karst cave, grounwater, lake, stratified lake;  methanotrophy in water column |
| 00007 | OP279927 | KP072474 | Gallionellaceae | 99,6 | 100 | A | A |  |  |  | O |  | R | groundwater, oligotrophic sediment, wetland sediment,  infiltration basin; habitats at redox transition zones |
| 00008 | OP279930 | CP016776 | Planktophila sp. | 99,6 | 100 | H | A |  |  |  |  |  | R | mounain lake, glacier seep, lake |
| 00011 | OP279931 | MN880334 | Glaciibacter sp. | 100 | 100 | H | A |  |  |  |  |  | R | ice wedge, cryoconite, permafrost, also irrelevant sites  (anaerobic digester) |
| 00012 | OP279932 | MN880330 | Pseudarcicella sp. | 100 | 100 | H | A |  |  |  |  |  | R | discovered in leech, psychrotolerant; water, dunite |
| 00013 | OP279933 | MN602482 | Methyloccocales | 100 | 100 | H | A |  |  | O |  |  | RM | lakes, Fe-rich snow, sediment at a methane seep |
| 00014 | OP279924 | MN880326 | Polaromonas sp. | 100 | 100 | F | A |  |  | O |  |  | R | soil, glacier, rain |
| 00015 | OP279935 | MZ569863 | Marinilabiliales | 100 | 100 | H | N |  |  |  |  |  | F | deglaciated sediment, fen, anaerobic digester; anaerobic sites |
| 00016 | OP279937 | MN733352 | Burkhorderiales | 99,6 | 100 | H | A |  |  |  |  |  | R | wastewater, forest soil, inundated soil, lake sediment, glacier surface,  mat; common, biofilm, rather eutrophic sites |
| 00017 | OP279938 | MN880328 | Luteolibacter sp. | 100 | 100 | H | A |  |  |  |  |  | R | soil, skin, lake, epilithic, insect gut; rather eutrohic sites |
| 00018 | OP279939 | MZ569866 | Anaerolineaceae | 100 | 100 | H | N |  |  |  |  |  | F | hydrocarbon contaminations, soil, bioreactor, parmafrost,  mountain glacier, deglaciated subglacial sediment |
| 00019 | OP279941 | MH463963 | Lysobacter sp. | 99,2 | 100 | H | A |  |  |  |  |  | R | glacier; proteolytic, lyses other microorg. |
| 00020 | OP279942 | MN072730 | Sedminibacterium | 99,6 | 100 | H | A |  |  |  |  |  | R | various freshwater sites (pond, river, drinkwater, thaw pond) |
| 00021 | OP279943 | CP016782 | Planktophila sp. | 98,8 | 100 | H | A |  |  |  |  |  | R | oligotrophic, auxotrophic; ocean crust and sediment,  hydrocarbon contaminations, methane cycling environments |
| 00022 | OP279944 | MN072727 | Polynucleobacter sp. | 100 | 100 | H | A |  |  |  |  |  | R | obligate symbiont of ciliates, freshwater |
| 00023 | OP279945 | MN880335 | Thiobacillus sp. | 100 | 100 | A | A |  | O |  |  |  | R | river, denitrifying env., hydrocarbon contaminations, sewer,  permafrost |
| 00026 | OP279946 | MN880354 | Pedobacter sp. | 100 | 100 | H | A |  |  |  |  |  | R | various soils, Antarctic coast, river |
| 00029 | OP279947 | KR854877 | Flectobacillus sp. | 99,2 | 100 | H | A | R |  |  |  |  | R | soil, river, river biofilm |
| 00030 | OP279948 | MN880348 | Aureivirga sp. | 100 | 100 | H | A |  |  |  |  |  | R | sponge, coal bed, aquifer, cont. groundwater, drinkw. |
| 00038 | OP279950 | KR842042 | Rhodobacteraceae | 99,6 | 100 | F | F | R | R |  |  | F | R | aerobic anoxygenic photosynthesis, carbon monoxide oxidation |
| 00040 | OP279951 | MN880350 | Pedobacter sp. | 100 | 100 | H | A |  |  |  |  |  | R | various soils, Antarctic coast, river |
| 00048 | OP279952 | KP785780 | Rhodobacteraceae | 98 | 100 | F | F |  |  |  |  | F | R | river sediment |
| 00070 | OP279929 | MZ569860 | Methylotenera | 100 | 100 | H | A |  |  |  |  |  | RM | eutrophic lake, parchment, sediments, biofilm, mine water;  eutrophic and redox boundary-related habitats |
| 00099 | OP279936 | AB836657 | Sphaerotilaceae | 100 | 100 | H | A |  |  |  |  |  | R | root nodules, water, skin, vagina, rapid filter, rhizosphere;  extremely common, mostly in trashy studies |
| 00171 | OP279953 | CP070630 | Dehalococcoidia | 96,8 | 100 | H | N |  |  |  |  |  | DHR | wetland, wastewater, coal seam; organic C-rich anaerobic habitats,  rare, only 3 blast hits over 99 % identity |
| 00215 | OP279934 | MT570022 | Methylotenera sp. | 100 | 100 | H | A |  |  |  |  |  | RM | spinach, roots, lava, mountain lake, hypolimnion,  under-ice methanotrophy; very common in both water and solids |
| 00418 | OP279949 | LT717416 | Limnohabitans sp. | 100 | 100 | H | A |  |  |  |  |  | R | freshwater habitats, bacterioplankton |
| 00789 | OP279940 | MN477136 | Limnohabitans sp. | 98,8 | 100 | H | A |  |  |  |  |  | R | lake, aquatic roots, amphipod, sponge; water biofilms,  not very common |
